# Immunoglobulin gene sequence analysis in chronic lymphocytic leukemia: the 2022 update of the recommendations by ERIC, the European Research Initiative on CLL

**DOI:** 10.1038/s41375-022-01604-2

**Published:** 2022-05-25

**Authors:** Andreas Agathangelidis, Anastasia Chatzidimitriou, Thomas Chatzikonstantinou, Cristina Tresoldi, Zadie Davis, Véronique Giudicelli, Sofia Kossida, Chrysoula Belessi, Richard Rosenquist, Paolo Ghia, Anton W. Langerak, Frédéric Davi, Kostas Stamatopoulos, on behalf of ERIC, the European Research Initiative on CLL

**Affiliations:** 1 Institute of Applied Biosciences, Centre for Research and Technology Hellas, Thessaloniki, Greece; 2 Department of Biology, School of Science, National and Kapodistrian University of Athens, Athens, Greece; 3 Department of Molecular Medicine and Surgery, Karolinska Institutet, Stockholm, Sweden; 4 Department of Hematology and HCT Unit, G. Papanicolaou Hospital, Thessaloniki, Greece; 5 Division of Immunology, Transplantation and Infectious Diseases, IRCCS Ospedale San Raffaele, Milan, Italy; 6 Department of Molecular Pathology, University Hospitals Dorset, Bournemouth, UK; 7 International ImMunoGeneTics Information System (IMGT), Institut de Génétique Humaine (IGH), Centre National de la Recherche Scientifique (CNRS), Université de Montpellier, Montpellier, France; 8 Hematology Department, Nikea General Hospital, Pireaus, Greece; 9 Clinical Genetics, Karolinska University Laboratory, Karolinska University Hospital, Stockholm, Sweden; 10 Division of Experimental Oncology, Università Vita-Salute San Raffaele and IRCCS Ospedale San Raffaele, Milan, Italy; 11 Department of Immunology, Laboratory Medical Immunology, Erasmus MC, Rotterdam, The Netherlands; 12 Department of Hematology, APHP, HôpitalPitié-Salpêtrière and Sorbonne University, Paris, France

**Keywords:** Translational research, Tumour immunology

## Abstract

The somatic hypermutation (SHM) status of the clonotypic immunoglobulin heavy variable (IGHV) gene is a critical biomarker for assessing the prognosis of patients with chronic lymphocytic leukemia (CLL). Importantly, independent studies have documented that IGHV SHM status is also a predictor of responses to therapy, including both chemoimmunotherapy (CIT) and novel, targeted agents. Moreover, immunogenetic analysis in CLL has revealed that different patients may express (quasi)identical, stereotyped B cell receptor immunoglobulin (BcR IG) and are classified into subsets based on this common feature. Patients in certain stereotyped subsets display consistent biology, clinical presentation, and outcome that are distinct from other patients, even with concordant IGHV gene SHM status. All of the above highlights the relevance of immunogenetic analysis in CLL, which is considered a cornerstone for accurate risk stratification and clinical decision making. Recommendations for robust immunogenetic analysis exist thanks to dedicated efforts by ERIC, the European Research Initiative on CLL, covering all test phases, from the pre-analytical and analytical to the post-analytical, pertaining to the analysis, interpretation, and reporting of the findings. That said, these recommendations apply to Sanger sequencing, which is increasingly being superseded by next generation sequencing (NGS), further underscoring the need for an update. Here, we present an overview of the clinical utility of immunogenetics in CLL and update our analytical recommendations with the aim to assist in the refined management of patients with CLL.

## Immunogenetic analysis in CLL: key to understanding and treating CLL

Immunogenetic studies have offered strong evidence for the central role of the B cell receptor immunoglobulin (BcR IG) in the natural history of chronic lymphocytic leukemia (CLL). Restrictions in the BcR IG gene repertoire, culminating in the existence of subsets with stereotyped BcR IG, strongly implicate antigen selection in CLL pathogenesis [1]. Of clinical relevance, the somatic hypermutation (SHM) status of the rearranged immunoglobulin heavy variable (IGHV) gene has emerged as key to accurate risk stratification in CLL [2]. Moreover, this biomarker remains stable over time, thus contrasting other, “cell-intrinsic” biomarkers, such as genomic aberrations, that are enriched in patients with advanced and/or relapsed/refractory disease [3].

On these grounds, it becomes apparent that robust immunogenetic characterization has an important role in the proper management of patients with CLL. This is reflected in the guidelines of the International Workshop on CLL (iwCLL) indicating that this biomarker should be assessed prior to treatment in all patients with CLL, i.e., in both general practice and clinical trials; [4] and, it has now been translated into clinical recommendations by many professional scientific societies worldwide, such as the National Comprehensive Cancer Network (NCCN) and the European Society for Medical Oncology (ESMO).

## IGHV gene somatic hypermutation status as a prognosticator in CLL

The prognostic value of SHM within the clonotypic rearranged IGHV genes was first recognized in 1999 [5, 6], when it was shown that patients with no or limited SHM (‘unmutated’ CLL, U-CLL) usually experience an aggressive form of CLL, while those with a significant SHM load (‘mutated’ CLL, M-CLL) follow more indolent disease courses [2]. Since then, many studies have confirmed these findings, rendering the analysis of IGHV gene SHM status an invaluable and non-dispensable tool for prognostication in CLL, regarding any relevant outcome measure.

Indeed, this biomarker may assist in predicting how soon patients will require treatment after the initial diagnosis, in other words, it can discriminate patients with shorter versus longer time-to-first-treatment (TTFT). Unsurprisingly, therefore, IGHV gene SHM status has been included in various prognostic tools/models/scores for TTFT, e.g., the CLL international prognostic index (CLL-IPI) [7], the CLL1 prognostic model [8], the International Prognostic Score for Early-stage CLL(IPS-E) [9] and the CLL WithOut Need of Treatment (CLL-WONT) risk score [10]. In both the CLL-IPI and the CLL1 model, unmutated IGHV gene status was given the highest score after del(17p), while in the IPS-E and CLL-WONT the presence of unmutated IGHV genes was independently associated with shorter TTFT [7, 10, 11]. Interestingly, the CLL-IPI and CLL-WONT were combined to identify patients with a very low risk of 5-year TTFT that can be initially managed by primary healthcare providers [10].

That notwithstanding, despite the strong prognostic value of IGHV gene SHM status at the cohort level, this test may not always be accurate at the individual case level. On the one hand, not all U-CLL patients will require treatment, while, on the other hand, some M-CLL cases will experience disease progression and need therapy. This finding raises doubts concerning the clinical utility of this information at the individual patient level at the time of diagnosis, before the development of any evidence of active disease.

Independent studies from the chemoimmunotherapy era have shown that U-CLL patients have a worse overall survival (OS) than M-CLL patients [12–16], with a meta-analysis of 13 studies published in 2016 reporting an OS hazard ratio of 1.6 to 6.9 for U-CLL patients [17]. M-CLL patients have been consistently found to fare better, except for two studies: more specifically, Shanafelt et al. showed that amongst M-CLL patients only those <75 years experienced an OS benefit [18], while in a small cohort by Ouillette et al. IGHV gene SHM status did not impact on OS [19].

## IGHV gene somatic hypermutation status as a predictor of responses to treatment in CLL

Results from prospective clinical trials have disclosed the predictive value of IGHV gene SHM status in patients with CLL who required treatment. Starting with chemoimmunotherapy, unmutated IGHV genes predicted for a worse progression-free survival (PFS). Specifically, in the CLL8 trial [20], which compared fludarabine, cyclophosphamide, and rituximab (FCR) versus fludarabine and cyclophosphamide (FC), where the presence of unmutated IGHV genes was predictive of worse PFS irrespective of the regimen used. In a longer follow-up of this study, M-CLL patients experienced better PFS and OS when treated with FCR compared with FC [21]. Similar results were reported by Thompson et al. [22]., who found a high rate of prolonged PFS in M-CLL patients treated with FCR; and, by Rossi et al. who documented a particularly good response to FCR in M-CLL patients lacking del(17p) or del(11q) [23].

The importance of IGHV gene SHM status was also highlighted in clinical trials of novel agents, such as BTK inhibitors (BTKis). In the RESONATE and RESONATE-2 trials, patients treated with ibrutinib, either in the front-line setting or for relapsed/refractory disease, experienced a better PFS and OS than patients treated with ofatumumab or chlorambucil, respectively [24, 25]. The superiority of BTKis over CIT was also documented in elderly patients and in the relapsed/refractory setting, more particularly the ALLIANCE (ibrutinib ±  rituximab versus bendamustine + rituximab) [26], ASCEND (acalabrutinib versus idelalisib + rituximab or bendamustine + rituximab) [27] and ILLUMINATE (ibrutinib + obinutuzumab versus chlorambucil + obinutuziumab) trials [28]. In those studies, the PFS benefit of BTKis was apparent, yet irrespective of the IGHV gene SHM status. However, in the ECOG 1912 (ibrutinib + rituximab (IR) versus FCR) [29] and ELEVATE-TN (acalabrutinib ± obinutuzumab versus chlorambucil + obinutuzumab) trials, the impact of U-CLL became apparent: in particular, U-CLL patients had a statistically superior PFS when treated with a BTKi based regimen compared with CIT, while the PFS difference for M-CLL patients was not significanty different [30]. Of note, longer follow-ups of RESONATE, RESONATE-2 and ELEVATE-TN trials showed no PFS difference between patients with M-CLL and U-CLL when treated with BTKis, highlighting the potential of this drug class to abrogate the prognostic impact of IGHV gene SHM status [27, 28, 30].

The relevance of IGHV gene SHM status has also been underscored by trials comparing the BCL2 inhibitor venetoclax plus anti-CD20 antibodies against CIT [31, 32]. Particularly for frontline treatment, in the CLL14 trial, U-CLL and M-CLL patients treated with venetoclax +  obinutuzumab for 12 months had a superior PFS compared to those treated with chlorambucil + obinutuzumab [33]. Interestingly, the PFS benefit for venetoclax + obinutuzumab appeared to be inferior for U-CLL versus M-CLL [34–36], raising the question as to whether fixed-duration treatment represents the optimal choice for U-CLL patients.

Altogether, immunogenetic analysis offers critical information that impacts the choice of treatment in CLL. A consensus exists that U-CLL patients have inferior outcomes when treated with CIT, therefore, these patients should preferentially be treated with novel agents [37].

## BcR IG stereotypy is clinically relevant: what is the evidence?

BcR IG stereotypy defines subsets of patients with consistent disease biology [38–44], clinical presentation, course, and outcome [1, 45, 46], including the response to therapy [11], at least for major subsets that have been the focus of most research. From a clinical perspective, the most notable examples are stereotyped subsets #2 and #8, both associated with aggressive disease [11, 45, 46]. Subset #2 includes patients with CLL expressing BcR IG encoded by the IGHV3-21/IGLV3-21 genes with distinctive, restricted VH and VL CDR3 sequences [1, 47–50], of whom the majority (~60–65%) carry somatically hypermutated IGHV genes. Subset #2 displays a remarkable enrichment for mutations of the *SF3B1* gene [38, 51, 52] and aberrations of the *ATM* gene, including both deletions and mutations [53]. Retrospective analyses of different cohorts of increasing size revealed that subset #2 patients experience a particularly aggressive disease course, irrespective of their IGHV gene SHM status [46–48, 54–57]. Notably, similar results were obtained in a meta-analysis of 3 prospective clinical trials conducted by the German CLL Study Group (CLL8: fludarabine-cyclophosphamide-rituximab versus fludarabine-cyclophosphamide; CLL10: fludarabine-cyclophosphamide-rituximab versus bendamustine-rituximab; CLL11: chlorambucil versus chlorambucil-rituximab versus chlorambucil-obinutuzumab). Membership of subset #2 was found to be an independent prognostic marker for shorter TTFT, time-to-next-treatment (TTNT), and PFS, irrespective of the SHM status [11]. These findings corroborate previous findings from the retrospective analysis of a large multi-institutional cohort of cases treated outside clinical trials where no improvement in OS was observed for subset #2 patients treated with CIT [57]. This evidence suggests that CIT may be less optimal for subset #2 patients, while also highlighting the usefulness of this information for risk stratification of patients, a practice already followed by different study groups worldwide. In that regard, it is relevant to mention the results of the NCRI FLAIR trial, where a hazard ratio for disease progression and death of 0.32 was reported for subset #2 patients treated with FCR versus IR, though not reaching statistical significance (*p* = 0.191), likely due to low numbers (FCR, *n* = 20; IR, n = 26) [58].

Subset #8 includes patients with CLL expressing BcR IG encoded by the IGHV4-39/IGKV1(D)-39 genes with distinctive, restricted VH and VK CDR3 sequences [54, 59]. The clonotypic IGHV genes are unmutated [54, 59], whereas, interestingly, all patients carry IgG-switched BcR [60], which is remarkable given their overalllow incidence in CLL (~8% of all cases). Subset #8 displays a significant enrichment for trisomy 12 [46] and *NOTCH1* mutations [38, 51, 52], offering yet another example of subset-biased profiles of genomic aberrations.

In the original report by Ghiotto et al. [59], it was noted that 2 of 5 patients belonging to subset #8 experienced Richter’s transformation. This initial observation was subsequently corroborated by a collaborative study from Italy, where it was reported that patients belonging to subset #8 had a 10-fold higher risk for this development compared to all other patients with CLL [45]. Subsequently, the meta-analysis of 3 prospective clinical trials conducted by the German CLL Study Group showed that patients belonging to subset #8 experienced the highest risk of Richter’s transformation, albeit the incidence was lower [11]. On these grounds, a closer follow-up and a lower threshold for a  fluorodeoxyglucose positron emission tomography (PET)/CT guided biopsy is warranted for subset #8 patients experiencing evidence of disease progression (also under therapy) in order to exclude the possibility of Richter’s transformation.

Assignment to subset #8 is also relevant from an immunogenetic standpoint, given the fact that the immunogenetic relation between the CLL and aggressive lymphoma clones represents the most important prognostic marker for cases with Richter syndrome (RS). More specifically, in the chemoimmunotherapy era, cases with immunogenetically unrelated RS clones exhibit a better prognosis (median survival of ∼5 years) compared to those with immunogenetically related RS clones (median survival of 8–16 months) [61, 62].

## Next-generation immunogenetic analysis in CLL: new possibilities, new challenges

Until a few years ago, Sanger sequencing was the only available methodology for immunogenetic studies in CLL, offering reliable information provided that rigorous standards recommended by ERIC, the European Research Initiative on CLL, were met [63]. The introduction of next-generation sequencing (NGS) allowed a far more detailed view of the BcR IG repertoires [64]. On the plus side, this added to our understanding of CLL [65–69]. On the minus side, several aspects of NGS in immunogenetics are not yet settled, especially in a diagnostic context. Indicatively, one could mention amplification biases and quantification issues as well as the paucity of multicenter-validated protocols. Another important consideration concerns the 98% germline identity cut-off, applied for discriminating cases into U-CLL and M-CLL categories [2]. This cut-off was decided through the study of Sanger-derived sequence data (i.e., low complexity) and its value has not been evaluated to the same degree in the NGS context (i.e., high complexity). Having said that, NGS immunogenetic analysis has not yet led to concrete evidence that would imply the need for reappraisal of the established 98% cut-off. Besides, employing NGS may cause issues of interpretation; indeed, NGS-based analysis can reveal the existence in the same patient of minor related clonotypes (corresponding to subclones arising due to intraclonal diversification) or unrelated clonotypes (corresponding to distinct clones) [64].

## Update of the ERIC recommendations for immunogenetic analysis in CLL

ERIC has issued recommendations on how to perform immunogenetic analysis in CLL that have been widely used by the CLL community [63, 70, 71]. Our accumulated knowledge and experience along with recent developments in the field prompted us to update these recommendations; the full list of our updated recommendations appears in Table 1. Selected topics are elaborated in more detail in the following paragraphs.

**Table 1:** Recommendations for the assessment of the somatic hypermutation status of the IGHV gene in clonotypic IGHV-IGHD-IGHJ gene rearrangements for standard (A) and difficult (B) cases in CLL.

*A. STANDARD CASES*	
Item	Recommendations
1. Methodology	Report type of: primers,^a^ PCR product analysis, sequencing method, bioinformatics tools for SHM status assessment, and stereotypy analysis.
2. IGH gene and allele identification	IGHV, IGHD^b^, IGHJ genes and alleles.
3. Functionality	SHM status determined only for productive rearrangements; if the rearrangement is unproductive, mention reasons for that (e.g., IG pseudogene, out-of-frame junction, stop codon, large indel).
4. IGHV gene: % of nucleotide identity to the germline to 2 decimal points as reported by IMGT	Classification: U-CLL ≥ 98%; M-CLL < 98%; borderline CLL when 97–97.99%.
5. Subset identification/BcR IG stereotypy	For subsets with well-established prognostic value (currently, subsets #2 and #8).
***B. CHALLENGING CASES***	
**Item**	**Recommendations**
1. Single unproductive rearrangement	Repeat the PCR with alternative primer sets and using cDNA. Perform NGS to get more detailed information regarding the clonal architecture. SHM status disclosed as not determined only in case all different approaches fail.
2. Double rearrangements	
2.1 One productive and one non-productive	Same as for standard cases: mutational status defined by the productive rearrangement, irrespective of the SHM status of the unproductive rearrangement.
2.2 Double productive	
2.2.1 Concordant SHM status	Same as for standard cases i.e., consider as M-CLL or U-CLL, according to the SHM status.
2.2.2 Discordant SHM status	Check immunophenotype for the presence of 2 clonal populations. Recommend to the physician that it is safer to consider as U-CLL; close follow-up.
3. Multiple (>2) productive rearrangements	Check immunophenotype for the presence of 2 or more clonal populations. Perform NGS to assess the relative frequency of each clonotype and consider the predominant clonotype, if it is clearly identified (NOTE: specific guidelines are still to be provided/developed here).
4. Missing anchors (C104/W118)	Mutational status assessment is possible if evidence for IG expression on leukemic cells and/or preserved G-X-G motif within the VH FR4.

^a^Leader primers are the only recommended option. That said, in rare cases when the application of a multiplex PCR with leader primers is unsuccessful VH FR1 primers can be used. The result should only be used to facilitate the application of a new round of PCR using IGHV subgroup-specific leader primers. Only if the result is suboptimal again, the report can be based on the VH FR1 PCR but it should be clearly stated that the use of VH FR1 primers might underestimate the total number of IGHV somatic hypermutations since a part of the VH domain is missing.

^b^In a percentage of cases, IGHD identification may be difficult due to: (i) excessive exonuclease trimming of the IGHD gene; and/or (ii) SHM within the VH CDR3, hindering the assignment to the closest germline IGHD gene and allele.

*CLL* chronic lymphocytic leukemia, *IG* immunoglobulin, *M-CLL* mutated CLL, *U-CLL* unmutated CLL.

Examples of lab report for both types of cases are provided in Supplementary Material.

### Standard cases

Standard cases characterized by the presence of a single productive IGHV-IGHD-IGHJ gene rearrangement should be analyzed according to the ERIC guidelines. Within this category, two major challenges are posed.

The first challenge concerns cases with borderline-mutated status i.e., those with a germline identity of 97–97.99%, for which we have already raised caution regarding the prognostic implications despite their formal assignment to the M-CLL category [71]. Recent studies showed that borderline-mutated cases either display a TTFT similar to that of M-CLL, excepting those belonging to subset #2 [72], or that the use of germline identy % as a continuous variable is associated with PFS and OS [73]. Against that however, accumulating evidence from the study of the borderline-mutated SHM subgroup revealed an enrichment of cases assigned to not only subset #2 but also its immunogenetic satellite, subset #169 [50], as well as other IGLV3-21 expressing cases, all harboring the R110 mutation [74–76]. This feature has emerged as adverse-prognostic irrespective of the BcR IGH rearrangement, underscoring the need to closely follow-up patients with borderline SHM status.

**Fig. 1:**
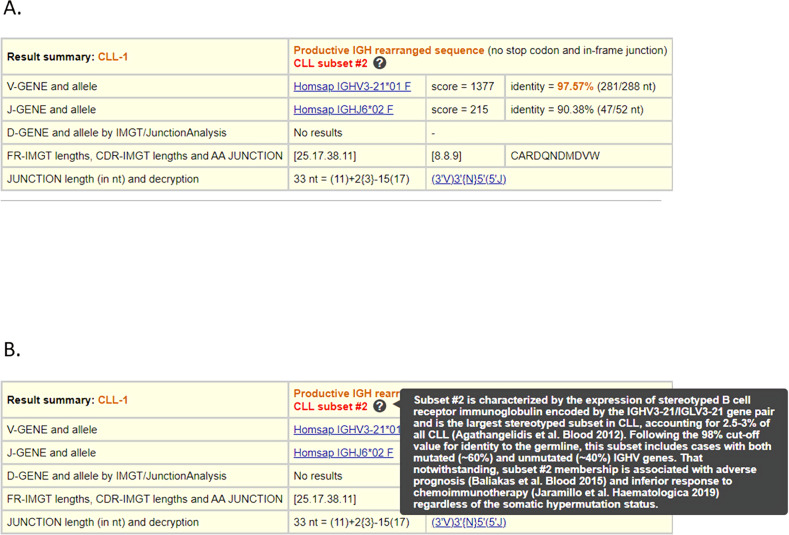
The advanced functionality “Clinical application: search for CLL subsets #2 or #8” incorporated in IMGT/V-QUEST. The functionality enables the identification of human IGH rearrangement sequences belonging to CLL subsets #2 or #8. The result is provided in the ‘Result summary’ after choosing the display results option ‘Detailed view’ (**A**). Evidence from relevant studies is also provided by the tool to assist the user (**B**).

The second challenge concerns BcR IG rearrangements belonging to the clinically aggressive stereotyped subsets #2 and #8 [1]. As already mentioned, subset #2 membership has emerged as an independent prognostic factor for inferior response to CIT and shorter TTNT, whereas membership in subset #8 has been associated with the highest risk for Richter’s transformation among all CLL. This information must be conveyed clearly to the physicians and included in the lab report [63]. Membership of either of these subsets can be investigated using either ARResT/AssignSubsets (http://bat.infspire.org/arrest/assignsubsets/) or IMGT/V-QUEST (http://www.imgt.org/IMGT_vquest/input), simply by selecting a new advanced functionality (Fig. 1).

### Challenging cases

#### Single unproductive rearrangements

IGHV-IGHD-IGHJ gene rearrangements can be rendered unproductive if they carry pseudogenes; out-of-frame VDJ junctions; stop codons; and/or indels leading to frameshifts within the coding part of the sequence. CLL cells are mature B cells that should express functional IG molecules on their surface. Hence, the identification of a single unproductive IGH rearrangement is exceedingly rare (<0.1% of all CLL) [71] and should always prompt further investigation in order to detect the productive IGHV-IGHD-IGHJ gene rearrangement on the other allele of the IGH locus. The possible mitigation steps when applying Sanger-based methodologies mainly concern the primers (utilize an alternative set), the starting material (try both gDNA and cDNA) and, occasionally, the sample (ask for a new sample). However, if all these approaches fail, NGS would be a reasonable alternative.

A similar issue may also arise with NGS in cases where the dominant clonotype is unproductive and coexists with one or more minor productive clonotypes. The most likely reasons for this finding are sequencing errors and/or amplification biases. In such cases, the analysis should be repeated; if the same result is obtained, one could attempt to PCR-amplify the unproductive as well as the most frequent productive rearrangements using IGHV- and IGHJ-gene specific primers and then apply bidirectional Sanger sequencing. If all the above strategies fail, single-cell analysis would be the only remaining approach. Admittedly, this is beyond the scope of a diagnostic lab and represents a research issue.

#### Double rearrangements: one productive and one unproductive

CLL cases with double rearrangements collectively account for 10.5% of all CLL [71]; within this category, the vast majority (~90% or 8.4% of all CLL) concern those cases with a productive and an unproductive rearrangement [71]. To date, there is no evidence supporting any kind of biological and/or clinical relevance for unproductive BcRIG gene rearrangements in CLL. Thus, the SHM status of such cases should be defined based solely on the productive IG rearrangement, irrespective of the SHM status of the unproductive rearrangement.

#### Double productive rearrangements with discordant mutational status

The identification of two, unrelated productive IGH rearrangements in cases with CLL could be due to the co-existence of two independent B cell clones: either a CLL clone and a separate, non-CLL B cell clone (i.e., a different malignancy) or two distinct CLL cell clones. Systematic immunophenotypic, molecular and morphological cell analysis may be required in order to make this distinction. In terms of prognosis, CLL cases with two B cell clones (a CLL and a non-CLL) have been reported to display earlier need for treatment against cases with monoclonal CLL; this may reflect a stronger clinical relevance of the other B cell malignancy compared to CLL [77]. In regard to cases with two unrelated CLL clones, if these clones present with concordant IGHV gene SHM status, they should be considered as either U-CLL or M-CLL, depending on the case.

A challenging scenario concerns cases carrying double productive rearrangements with discordant IGHV gene SHM status (<0.1% of all CLL) [4]. Evidence from low-throughput analysis [78] supports the notion that, in most cases, multiple productive IGH rearrangements in CLL derive from independent clones and display clonal drift, given the change in their relative frequencies observed over time. From a clinical perspective, these cases were found to exhibit adverse prognostic markers and aggressive disease, presenting with early need for treatment, similar to patients with U-CLL [78]. Evidence from NGS studies [68, 69] further supported the existence of biclonal cases in CLL. Interestingly, single-cell analysis showed that minor IG rearrangements can persist over time and may account for a sizeable fraction of the total repertoire (frequency range: 0.2–3%), indicating their biological and, perhaps, clinical relevance [68].

In this context, immunophenotypic analysis should be performed in order to verify the presence of the two B cell clonal populations. If their presence is verified, we recommend that both IGH gene rearrangements are reported to the physician. Regarding the clinical implications, the difficulty in providing a definitive assignment into one SHM category should be acknowledged. That said, erring on the side of benefit for the patient, we would propose/favor to manage these patients as U-CLL.

#### Multiple (3 or more) productive rearrangements

Patients carrying multiple (3 or more) IGH gene rearrangements were particularly scarce with Sanger analysis. Using NGS, multiple clonotypes can be detected in a varying fraction of cases: in most of these, multiple secondary clonotypes persisted over a significant period of time without major changes against the primary CLL clonotype [68, 69]. Having said that, a proper cut-off value for discriminating between secondary leukemic expansions, immune reactive cell clones and the normal “background” has not been established. Careful examination of the immunophenotypic results is strongly warranted in order to verify the presence of actual leukemic or reactive cell clones. To date, the evidence regarding the true meaning of multiple co-existing, immunogenetically unrelated, clonotypes remains inconclusive.

As above, we suggest that all verified IGH gene rearrangements are reported to the physician, including their relative frequencies. The difficulty in categorizing such cases when their SHM statusis discordant should be acknowledged. However, we would suggest to manage such cases as U-CLL, particularly if the dominant clonotype belongs to this category.

#### Concluding remarks

Immunogenetic analysis is key to understanding and managing CLL. Relevant procedures are standardized end-to-end, allowing to offer robust and accurate information in both general practice and clinical trials. As for any other laboratory test, challenging cases will always exist, albeit very infrequently: in such cases, careful assessment of all pertinent information is warranted before reaching conclusions. Any scientist and/or physician who would like to request assistance should also be aware of the dedicated online troubleshooting service by ERIC (https://barcelo.eventsair.com/submission-of-ighv-sequences/ighv-sequences/Site/Register) who can offer expert guidance and suggestions for overcoming the challenge.

ERIC will continue its efforts to facilitate the standardization of immunogenetic analysis in CLL, through accumulating knowledge from scientific discoveries as well as experience from the application of novel NGS methodologies.

## Supplementary information

Supplementary material
