# Supplementary material for: Immunoglobulin gene sequence analysis in chronic lymphocytic leukemia: the 2022 update of the recommendations by ERIC, the European Research Initiative on CLL
Source: Leukemia. 2022 May 25;36(8):1961–8. doi: 10.1038/s41375-022-01604-2 (PMC9343247; doi:10.1038/s41375-022-01604-2)
Supplement: Supplementary file 1 — Supplementary material [file 41375_2022_1604_MOESM1_ESM.docx]

##### *1. Example of the IG report, IG - mutated*

##### Name of the Hospital/Lab

**Determination of IGHV gene SHM status**

Date of result: 22/01/2022

Date of sample collection: 09/01/2022

**Patient name:** ***

Diagnosis: CLL

Tissue type: blood

Molecule type: genomic DNA

**Utilized methodology**

PCR amplification of IGHV-IGHD-IGHJ gene rearrangements with leader primers.

Genescan analysis

Bidirectional Sanger sequencing

Immunoinformatics analysis: IMGT/V-QUEST

**Result:** a productive IGHV3-23*01/IGHD4-17*01/IGHJ4*02 gene was detected. The rearranged IGHV gene had 96.2% nucleotide identity with the germline sequence of the IGHV3-23*01 gene.

**Interpretation:** following the 98% germline identity cut-off value which is used for discriminating CLL cases into the IG-mutated or IG-unmutated category, this case belongs to the IG-mutated category which is generally associated with favorable prognosis.

Signatures

##### *2. Example of the IG report, IG - unmutated*

##### Name of the Hospital/Lab

**Determination of IGHV gene SHM status**

Date of result: 22/01/2022

Date of sample collection: 09/01/2022

**Patient name:** ***

Diagnosis: CLL

Tissue type: blood

Molecule type: genomic DNA

**Utilized methodology**

PCR amplification of IGHV-IGHD-IGHJ gene rearrangements with leader primers.

Genescan analysis

Bidirectional Sanger sequencing

Immunoinformatics analysis: IMGT/V-QUEST

**Result:** a productive IGHV3-49*01/IGHD3-9*01/IGHJ4*02 gene was detected. The rearranged IGHV gene had 100% nucleotide identity with the germline sequence of the IGHV3-49*01 gene.

**Interpretation:** following the 98% germline identity cut-off value which is used for discriminating CLL cases into the IG-mutated or IG-unmutated category, this case belongs to the IG-unmutated category which is generally associated with adverse prognosis and poor response to chemo(immuno)therapy.

Signatures

##### *3. Example of the IG report, IG - borderline mutated*

##### Name of the Hospital/Lab

**Determination of IGHV gene SHM status**

Date of result: 22/01/2022

Date of sample collection: 09/01/2022

**Patient name:** ***

Diagnosis: CLL

Tissue type: blood

Molecule type: genomic DNA

**Utilized methodology**

PCR amplification of IGHV-IGHD-IGHJ gene rearrangements with leader primers.

Genescan analysis

Bidirectional Sanger sequencing

Immunoinformatics analysis: IMGT/V-QUEST

**Result:** a productive IGHV3-49*01/IGHD3-9*01/IGHJ4*02 gene was detected. The rearranged IGHV gene had 97.3% nucleotide identity with the germline sequence of the IGHV3-49*01 gene.

**Interpretation:** following the 98% germline identity cut-off value which is used for discriminating CLL cases into the IG-mutated or IG-unmutated category, this case belongs to the IG-mutated category. However, the identity percentage is close to the 98% cut-off and, thus, the case can be considered as borderline-mutated. In such cases, caution is warranted regarding the precise prognostic implications.

Signatures

##### *4. Example of the IG report, IG - subset #2*

##### Name of the Hospital/Lab

**Determination of IGHV gene SHM status**

Date of result: 22/01/2022

Date of sample collection: 09/01/2022

**Patient name:** ***

Diagnosis: CLL

Tissue type: blood

Molecule type: genomic DNA

**Utilized methodology**

PCR amplification of IGHV-IGHD-IGHJ gene rearrangements with leader primers.

Genescan analysis

Bidirectional Sanger sequencing

Immunoinformatics analysis: IMGT/V-QUEST

**Result:** a productive IGHV3-21*01/IGHD: not determined/IGHJ6*02 gene was detected. The rearranged IGHV gene had 96.8% nucleotide identity with the germline sequence of the IGHV3-21*01 gene.

**Interpretation:** following the 98% germline identity cut-off value which is used for discriminating CLL cases into the IG-mutated or IG-unmutated category, this case belongs to the IG-mutated category. However, this particular rearrangement belongs to stereotyped subset #2 which is associated with adverse prognosis and poor response to chemo(immuno)therapy regardless of the somatic hypermutation status (Baliakas et al. Blood 2015; Jaramillo et al. Haematologica 2020).

Signatures

##### *5. Example of the IG report, IG - subset #8*

##### Name of the Hospital/Lab

**Determination of IGHV gene SHM status**

Date of result: 22/01/2022

Date of sample collection: 09/01/2022

**Patient name:** ***

Diagnosis: CLL

Tissue type: blood

Molecule type: genomic DNA

**Utilized methodology**

PCR amplification of IGHV-IGHD-IGHJ gene rearrangements with leader primers.

Genescan analysis

Bidirectional Sanger sequencing

Immunoinformatics analysis: IMGT/V-QUEST

**Result:** a productive IGHV4-39*01/IGHD6-13*01/IGHJ5*02 gene was detected. The rearranged IGHV gene had 100% nucleotide identity with the germline sequence of the IGHV4-39*01 gene.

**Interpretation:** following the 98% germline identity cut-off value which is used for discriminating CLL cases into the IG-mutated or IG-unmutated category, this case belongs to the IG-unmutated category. Furthermore, this particular rearrangement belongs to stereotyped subset #8 which is associated with the highest risk for Richter’s transformation among all CLL (Rossi et al. Clinical Cancer Research 2009).

Signatures
